# Metabolic and Immune Parameters in Pregnant Women with Impaired Glucose Metabolism—A Pilot Study

**DOI:** 10.3390/metabo14100551

**Published:** 2024-10-16

**Authors:** Jelena Omazić, Andrijana Muller, Blaž Dumančić, Mirta Kadivnik, Jasna Aladrović, Lana Pađen, Kristina Kralik, Nikolina Brkić, Blaženka Dobrošević, Barbara Vuković, Jasenka Wagner

**Affiliations:** 1 Department of Laboratory and Transfusion Medicine, “Dr. Juraj Njavro” National Memorial Hospital, 32000 Vukovar, Croatia; jelena.omazic@gmail.com; 2 Department of Medical Chemistry, Biochemistry and Clinical Chemistry, Faculty of Medicine, J.J. Strossmayer University, 31000 Osijek, Croatia; 3 Clinic of Obstetrics and Gynecology, University Hospital Center Osijek, 31000 Osijek, Croatia; 4 Department of Obstetrics and Gynecology, Faculty of Medicine, J.J. Strossmayer University, 31000 Osijek, Croatia; 5 Department of Medical Biology and Genetics, Faculty of Medicine, J.J. Strossmayer University, 31000 Osijek, Croatia; 6 Department of Physiology and Radiobiology, Faculty of Veterinary Medicine, University of Zagreb, 10000 Zagreb, Croatia; 7 Department of Medical Statistics and Informatics, Faculty of Medicine, J.J. Strossmayer University, 31000 Osijek, Croatia; 8 Department of Transfusion Medicine, General Hospital Vinkovci, 32100 Vinkovci, Croatia; 9 Institute of Clinical Laboratory Diagnostics, University Hospital Centre Osijek, 31000 Osijek, Croatia; 10 Poliklinika Slavonija, 31000 Osijek, Croatia

**Keywords:** gestational diabetes, pregnancy, glucose, glucose intolerance, lymphocytes, fatty acids

## Abstract

Gestational diabetes mellitus (GDM) is a public health problem with increasing prevalence. Analyses of metabolic and immune profiles have great potential for discovering new markers and mechanisms related to the development of GDM. We monitored 61 pregnant women during the first and third trimesters of pregnancy, including 13 pregnant women with GDM, 14 pregnant women with elevated glucose in the first trimester and 34 healthy pregnant women. A number of metabolic and immunological parameters were measured, including glucose, insulin, lipid status, fatty acids, lymphocyte profile, adiponectin, IL-6, IL-10 and TNF-a. A higher number of T-helper lymphocytes and a higher ratio of helper/cytotoxic lymphocytes was found in the control group in the first trimester of pregnancy. Pregnant women whose glucose threshold values were measured in the first trimester, but who did not develop GDM, showed a higher percentage of neutrophils and a lower percentage of lymphocytes in the third trimester. Differences in polyunsaturated fatty acids levels were observed between healthy pregnant women and those with glucose metabolism disorders in the first trimester of pregnancy. The results of this pilot study demonstrate that there are differences in the profiles of T lymphocytes, NK cells and polyunsaturated fatty acids between the examined groups of pregnant women, which can serve as a direction for future research.

## 1. Introduction

Gestational diabetes mellitus (GDM) is a condition characterised by abnormal glucose tolerance, which appears during pregnancy and usually disappears after the delivery [1]. Its prevalence is increasing along with increases in the prevalence of obesity and type 2 diabetes mellitus [1]. A large survey of the global prevalence of GDM during the year 2021 showed a prevalence of 14.0%. According to research data, the highest prevalence of 27.6% was observed in the Middle East and North Africa, followed by 20.8% in Southeast Asia, 14.7% in the Western Pacific and 14.2% in Africa. In South and Central America, the prevalence was 10.4%, while in North America and the Caribbean, it was 7.1%. The prevalence in Europe in 2021 was 7.8% [2]. Gestational diabetes mellitus is the most common metabolic disorder in pregnancy, not only affecting pregnancy and childbirth but also potentially leading to long-term consequences for the mother and the foetus [3]. Although the first records of gestational diabetes date back to the 19th century, the pathophysiology of gestational diabetes is still not fully understood [4]. There are several known risk factors for the development of gestational diabetes, including maternal age, polycystic ovary syndrome, foetal macrosomia in a previous pregnancy, obesity, family history of diabetes and significant weight gain during pregnancy [5]. It has been reported that 35% of women who have had GDM will develop diabetes during their lifetime [6].

Pregnancy is a state characterised by physiological insulin resistance, which primarily develops under the influence of placental hormones (e.g., human placental lactogen, progesterone, oestrogen), as well as prolactin and cortisol. It grows slowly during pregnancy, reaches its peak between the 24th and 28th weeks and then stabilises. Diabetic pregnant women have a higher degree of insulin resistance compared to the physiological resistance that occurs during pregnancy [7]. Due to the insulin resistance of the peripheral tissues, the pancreas compensates by producing more and more insulin, which results in beta-cell hypertrophy and hyperinsulinemia. Beta cells are thought to fail due to excessive insulin production in response to peripheral tissue resistance, which eventually depletes them [8]. 

Analyses of immune profiles have great potential for discovering new markers and mechanisms related to insulin resistance and the development of GDM [9,10]. The immune system protects an organism by distinguishing foreign substances from its own. It has two parts: innate and acquired immunity. Lymphocytes are immune cells that recognise and respond to foreign substances, of which there are three types: T lymphocytes, B lymphocytes, and natural killer (NK) cells. These cells can be identified using flow cytometry. T and B lymphocytes are essential for immune responses. Cytotoxic T lymphocytes destroy target cells, while helper T lymphocytes coordinate other immune cells. B lymphocytes help to regulate inflammation in fat tissue. NK cells are a type of T cell that help to regulate immune responses, including those involved in obesity and diabetes [11,12,13,14]. GDM is characterised by an abnormal immune response, often accompanied by low-grade inflammation and negative health consequences for the mother. This inflammatory response stems from disruptions within the maternal immune system. Both innate and adaptive immune cells are involved, with excessive tissue infiltration and heightened activation in response to high glucose and insulin resistance, leading to the release of various inflammatory markers. However, the specific immune cell types driving the pathology of GDM remain an area of active research [13]. 

GDM is usually diagnosed during the second trimester of pregnancy, according to the recommendations of the International Association of Diabetes and Pregnancy Study Groups (IADP-SG) [15]. According to these studies, fasting glucose or glycosylated haemoglobin (HbA1c) testing should be performed for all pregnant women during the first trimester of pregnancy. Pregnant women with normoglycemia should carry out an oral glucose tolerance test (OGTT) between weeks 24 and 28 of pregnancy in order to diagnose gestational diabetes. Gestational diabetes is diagnosed if glucose in the fasting OGTT is ≥5.1 mmol/L, ≥10 mmol/L after the first hour or ≥ 8.5 mmol/L after the second hour.

Given that the pathophysiology of GDM and the immunological and metabolic changes in pregnant women with GDM have yet to be fully investigated and elucidated, we aimed to examine and compare the metabolic and immune profiles in the first and third trimesters of pregnancy between pregnant women with GDM, healthy pregnant women and pregnant women with fasting glucose disorder in the first trimester. The aim of our research was to compare the differences between the mentioned groups of pregnant women in terms of their T and B lymphocyte profiles, including subpopulations of B lymphocytes with regard to the CD5+ marker, differences in the secretion of cytokines and adiponectin, and the profile of fatty acids. In the existing literature, we did not find a single study that included an examination of T and B lymphocytes, as well as the subpopulation of B lymphocytes with regard to the CD5+ marker. This research is a pilot study, through which we aimed to investigate whether the mentioned parameters can help improve our understanding of the pathophysiology of GDM and if there is a marker that can indicate whether a pregnant woman in the first trimester of pregnancy will develop GDM.

## 2. Materials and Methods

This prospective cohort study included pregnant women recruited in the first trimester of pregnancy (8–12 weeks of gestation), who were monitored throughout their pregnancy. OGTT was performed between 24 and 28 weeks of gestation, and additional data and blood samples were also taken from them in the third trimester of pregnancy (30–40 weeks), when all metabolic and immune profile analyses conducted in the first trimester of pregnancy were performed again. The main criterion used to select the pregnant women was that they were currently in good general health and had no known immunological diseases. The exclusion criteria were failure to sign informed consent, diagnosis of diabetes outside pregnancy and other metabolic diseases outside pregnancy.

Gestational age was determined using the first day of the last menstrual cycle and confirmed by early ultrasound. If there was a discrepancy, the ultrasound finding was used. The study was conducted at the Clinic of Gynecology and Obstetrics and Department of Clinical Laboratory Diagnostic of the University Hospital Centre Osijek, the Department for Laboratory and Transfusion Medicine of the “Dr. Juraj Njavro” National Memorial Hospital Vukovar and the Department of Medical Biology and Genetics of the Faculty of Medicine Osijek between February 2022 and December 2023, in accordance with the standards set by the last revision of the Declaration of Helsinki [16]. The research protocol and procedures were approved by the Ethics Committees of all institutions involved in the research. Written informed consent was obtained from all participants and their anonymity was guaranteed.

### 2.1. Participants

The study enrolled 61 pregnant women, who were divided into three groups in the third trimester of pregnancy after OGTT: 13 pregnant women with gestational diabetes, 14 pregnant women who were diagnosed with a glucose disorder in the first trimester (based on the results of fasting blood glucose measured in the first trimester and the OGTT result) and 34 healthy pregnant women as a control group. Pregnant women with normal glucose and HbA1c levels, no gestational diabetes or other pregnancy pathologies, a normal OGTT result, no family history of diabetes and no known autoimmune or metabolic diseases outside of pregnancy were included in the control group of participants. Pregnant women diagnosed with GDM during the current pregnancy according to the IADP-SG guidelines were included in the group of pregnant women with GDM. Pregnant women with glucose (5.1 mmol/L or higher) levels in the first trimester and normal OGTT results were included in the third group of participants. 

### 2.2. Blood Sampling

Each pregnant woman had her blood collected, in fasting state, in the morning between 8 and 10 a.m., in the first and third trimesters of pregnancy. In the first trimester, we collected three tubes of peripheral venous blood in a BD Vacutainer (BD, NJ, USA) with anticoagulant K3EDTA and one serum tube of peripheral venous blood in a BD Vacutainer (BD, NJ, USA) from each pregnant woman. In the third trimester, we collected two tubes of peripheral venous blood in a BD Vacutainer (BD, NJ, USA) with anticoagulant K3EDTA and one serum tube of peripheral venous blood in a BD Vacutainer (BD, NJ, USA) from all subjects. Analyses of samples of fresh whole blood, serum and plasma were performed. Serum and plasma were prepared by centrifuging a serum tube and one K3EDTA tube at a speed of 3000 g for 10 min at room temperature. 

### 2.3. Routine Biochemical and Haematological Analyses

Glucose levels were determined according to the enzymatic method using hexokinase on a cobasPro (Roche Diagnostic, Basel, Switzerland) analyser. Using the same analyser, total cholesterol, HDL and LDL cholesterol, triglycerides, high-sensitivity CRP (hs-CRP), immunoglobulins (class IgG, IgM and IgA), HbA1C, interleukin (IL)-6, insulin and C-peptide were determined. Homeostatic Model Assessment for Insulin Resistance (HOMA-IR) was performed using the glucose and insulin levels measured for all participants. The total blood count was determined using an Advia 2120i analyser (Siemens Healthineers, Erlangen, Germany). Lymphocyte, NK and NKT cells from the whole blood samples of pregnant women were analysed via flow cytometry (cytometer BD LSR II with acquisition software BD FACSDiva 8.0.1. and FloJo analysis, BecktonDickinson, Germany), by combining the surface cell markers CD3+, CD5+, CD3+CD4+, CD3+CD8+, CD3-CD16+CD56+, CD3+CD16+CD56+, CD19+CD20+ and CD19+CD5+, which define individual leukocyte subpopulations and determine their functions. Surface markers for lymphocytes, NK cells and NKT cells were analysed using commercial reagents containing specific monoclonal antibodies labelled with fluorochrome (Beckton Dickinson, Germany): BD Multitest CD3 FITC/CD8 PE/CD45 PerCP/CD4 APC and BD Multitest CD3 FITC/CD16 + 56 PE/CD45 PerCP/CD19 APC.

### 2.4. ELISA Tests

IL-10, tumour necrosis factor alpha (TNF-α) and adiponectin concentrations were determined using commercial ELISA tests from serum samples that had been stored at −70 °C from the time of sampling until analysis. For IL-10, we used a Human IL-10 ELISA Kit (Millipore, Sigma-Aldrich, Inc., St. Louis, MA, USA); for TNF-a, we used a Human TNF alpha SimpleStep ELISA Kit (abcam, Cambridge, UK); and, for adiponectin, we used a Human ADP ELISA Kit (ELK Biotechnology, Denver, CO, USA). 

### 2.5. Determination of Fatty Acids

Fatty acids were determined from EDTA plasma samples that had been stored at −70 °C immediately after sampling until analysis. Total lipids were extracted using a mixture of chloroform/methanol (2:1 *v*/*v*) via a modified method according to FOLCH et al. [17]. Lipid extracts were concentrated in a tabletop Centrivap vacuum concentrator, equipped with a Centrivap cooling unit and a diaphragm vacuum pump at 230 V and 50/60 Hz (Labconco, Kansas City, MO, USA). Fatty acids (FAs) from the total lipid extract were converted to methyl esters (FAMEs) via transesterification with methanolic HCl, according to international standard procedure ISO 5509 (2000). 

Analysis of FA methyl esters was performed on a gas chromatograph (Agilent 8860; Agilent Technologies. Inc., Sancta Clara, CA, USA) equipped with a flame ionisation detector (FID). The temperatures of the injector and detector were 200 °C and 240 °C, respectively. Chromatography was performed on a DB-23 capillary column (Agilent Technologies, Santa Clara, CA, USA) with length of 60 m, inner column diameter of 0.25 mm and active layer thickness of 0.25 μm. The temperature regime was as follows: 150 °C for 2 min, increased to 230 °C by 5 °C/min, and held for 20 min. Hydrogen at a flow rate of 1 mL/min was used as the carrier gas. The results were processed using the computer programme OpenLAB CDS ChemStation, Workstation VL. FAMEs were identified by comparing retention times with methyl standards (Sigma Aldrich Chemie, GmbH, Taufkirchen, Germany and Supelco, Bellefonte, PA, USA). Quantification was performed using nonadecanoic acid methyl ester (C19:0). The fatty acid composition was calculated as the percentage of each individual FA relative to the total FA. FAMEs were categorised according to their chain length and structure; namely, they were categorised as saturated (SFA) if they did not contain any unsaturated double bond and any methyl branches; as monounsaturated (MUFA) if they contained one double bond; and as polyunsaturated (PUFA) if they contained more than one double bond.

Stearoyl-CoA desaturase (SCD) activity indices were estimated by computing the ratio of product/(substrate + product) in plasma: SCDi-16, (16:1/(16:1 + 16:0)100); SCDi-18, (18:1/(18:1 + 18:0)100).

### 2.6. Statistical Analysis

The statistical programme MedCalc^®^ Statistical was used for data analysis (Software version 20.218; MedCalc Software Ltd., Ostend, Belgium; https://www.medcalc.org; accessed on 1 July 2024). Categorical data are represented by absolute and relative frequencies. The normality of the distribution of numerical variables was tested with the Shapiro–Wilk test and, due to the non-normality of the distribution, the data are described using the median and the limits of the interquartile range. Differences in categorical variables were tested with Fisher’s exact test. Differences in numerical variables between two independent groups were tested with the Mann–Whitney U-test. Non-parametric statistical tests, including the Friedman test and Kruskal–Wallis test, were used to compare the values obtained at the measurement points within and between the examined groups. All *p* values were two-sided. The significance level was set at 0.05.

## 3. Results

### 3.1. General Characteristics and Lifestyle of Pregnant Women

The general characteristics and lifestyles of the pregnant women in the individual groups are detailed in Table 1. No statistically significant differences were observed between the three groups of participants in terms of age, BMI, waist-to-hip ratio, eating habits and physical activity and general health status.

### 3.2. Past Gynaecological and Obstetric Anamnesis

Looking at the gynaecological and obstetric anamnesis (Table 2), a statistically significant difference was found in the weight of the newborn for the previous pregnancy in the group of pregnant women who developed GDM, compared to the control group and the group of pregnant women with glucose disorders at the beginning of pregnancy (*p* = 0.03).

### 3.3. Metabolic and Immunological Parameters in Pregnancy 

A statistically significant difference was found in all points of the OGTT, both in pregnant women with GDM and in pregnant women with glucose disorders at the beginning of pregnancy, compared to the control group of pregnant women (Table 3). 

Differences were also observed in the weight obtained between the two blood sampling time points in both groups. Pregnant women with GDM had the least difference in body weight, while pregnant women with glucose disorders at the beginning of pregnancy gained the most weight between the two blood samplings.

The results of the flow cytometry analysis indicate that the control group of healthy pregnant women had more T-helper lymphocytes and a higher ratio of helper/cytotoxic lymphocytes compared to the other two investigated groups in the first trimester of pregnancy. In the third trimester, statistically significant differences between groups were observed only in hsCRP and iron.

Statistical analysis of the obtained results showed that, in healthy pregnant women, there were statistically significant differences in erythrocytes, platelets, leukocytes, the percentage of T lymphocytes, T-helper lymphocytes, the ratio of helper and cytotoxic lymphocytes, NK cells and the percentage of B lymphocytes and CD 5+ and CD5- B lymphocytes between the first and third trimester. Statistically significant differences were also found in the obtained values of insulin, C peptide, HOMA index, HbA1c and immunoglobulin G. Similar results were obtained in pregnant women with GDM; however, in that group of subjects, there were no statistically significant differences in B lymphocytes and CD5+ and CD5- B lymphocytes. Pregnant women who had their glucose threshold values measured in the first trimester, but did not develop GDM, showed a higher percentage of neutrophils and a lower percentage of lymphocytes in the third trimester compared to the first trimester. As in pregnant women who developed GDM, there was no statistically significant difference in the percentage of B lymphocytes; however, a difference was found in the comparison of CD5+ and CD5- lymphocytes between the first and third trimesters. In these pregnant women, a statistically significant difference was obtained in the measured glucose values between the first and third trimesters, as well as in the inflammatory parameters hsCRP and immunoglobulins G and M (Table 4, Table 5).

Analysis of fatty acids indicated statistically significant differences in MUFAs, PUFAs, SFAs and unsaturated fatty acids (UFAs) between pregnant women in the first and third trimesters. When only the first trimester was considered, a statistically significant difference was found in PUFA between pregnant women with GDM and those with impaired fasting glucose in the first trimester compared to healthy pregnant women. The ratio of PUFAs and SFAs was statistically different only between healthy subjects and subjects with impaired fasting glucose in the first trimester (Table 6). 

## 4. Discussion

In our study, there were no statistically significant differences in between the examined groups with regard to the general characteristics and lifestyles of pregnant women; in particular, no statistically significant difference was observed in terms of age, BMI, waist-to-hip ratio, eating habits and physical activity and general health. Unlike in our study, in most other studies there was a statistically significant difference in BMI between healthy pregnant women and pregnant women who developed GDM, with pregnant women with GDM having a higher BMI [18,19,20]. Therefore, high BMI in early pregnancy is associated with altered lipid metabolism that may contribute to an increased risk of GDM. Mirabelli et al. proved, in their study, that the BMI of the mother prior to pregnancy plays a key role in determining GDM. BMI is an indicator of visceral obesity and systemic insulin resistance and is a modifiable risk factor for GDM; therefore, the authors of the study suggested it as a suitable target for raising public awareness about GDM [21].

We found statistically significant differences in all points of the OGTT, both in pregnant women with GDM and in pregnant women with glucose disorders at the beginning of pregnancy compared to the control group of pregnant women. As expected, a study that involved a significantly larger population of pregnant women (i.e., 690 participants) showed similar results regarding fasting plasma glucose and glucose levels after the OGTT load, where plasma glucose after 1 and 2 h was significantly higher in the GDM group than in the group of healthy subjects [22]. In our study, pregnant women from the group with GDM had significantly higher values for all three points of the OGTT, which is to be expected from a physiological standpoint, considering that they have impaired insulin resistance and β-cell function of the pancreas and, for this reason, they developed diabetes during pregnancy. Pregnant women who had a fasting glucose value of 5.1 mmol/L or higher in the first trimester also had higher fasting glucose values than the control group of pregnant women during the OGTT. In those pregnant women, the second measurement point (an hour after ingestion of the solution) was also higher than the control group of pregnant women, which may indicate that the examined group of pregnant women has a glucose metabolism disorder that did not manifest as GDM.

Statistical analysis of the obtained results showed that, in healthy pregnant women, there are statistically significant differences in erythrocytes, platelets, leukocytes, the percentage of T lymphocytes, T-helper lymphocytes, the ratio of helper and cytotoxic lymphocytes, NK cells and the percentage of B lymphocytes and CD 5+ and CD5- B lymphocytes between the first and third trimesters. In a large study conducted over six years ago, Zhang et al. obtained similar results for haematological parameters, including an increase in the number of leukocytes and a decrease in the number of platelets and erythrocytes over the course of pregnancy (which was more pronounced in GDM) [23]. Fashami et al. reported an association between the risk of developing GDM and parameters of the red blood count, where the highest association with an increased risk for GDM was observed for an elevated haematocrit level in the second trimester [24]. Yang et al. have reported that elevated numbers of erythrocytes, leukocytes and platelets are associated with GDM, and that a blood count may be considered as a low-cost prenatal test for GDM. Unfortunately, our research included a much smaller number of subjects than the study of Yang et al. (which included 614 women), and we did not observe a difference in the parameters of the blood count [25]. Sissala et al., in their study on 1828 pregnant women with GDM, observed higher haemoglobin values in GDM, and so they reported higher haemoglobin levels in the mother as an independent risk factor for GDM; however, they also reported that this has little effect on the perinatal outcome [26]. In our study, there was no statistically significant difference in haemoglobin values between healthy pregnant women and pregnant women with GDM, although pregnant women with GDM had higher haemoglobin values, as previously reported. Mehrabian and Hosseini have also claimed that high haemoglobin in the first trimester is associated with a higher risk of pre-eclampsia and GDM [27]. It is possible that we did not find a statically significant difference due to the small number of subjects in the study population, especially in the group of pregnant women with GDM.

Several other studies have linked increased WBC, neutrophils, monocytes and an increased neutrophil–lymphocyte ratio (NLR ratio) in early and mid-pregnancy and their high levels during pregnancy with a higher risk of GDM [28,29,30,31]. Researchers have found that an elevated neutrophil count in the first trimester of pregnancy is an independent risk factor and predictive factor for GDM. However, the relationship between lymphocytes and GDM is much more controversial, and NLR is considered as the best indicator as it is less affected by infection and stress, and as it is an indicator of inflammation and immune regulation [32]. An increase in NLR in diabetics may be a consequence of a decrease in the number of lymphocytes and a lack of immune regulation [33]. Our study did not show differences in the number of neutrophils, either in the first or third trimester, between the control group and group of patients with GDM, while previous studies showed a strong correlation between the number of neutrophils and the risk of developing GDM [33]. However, in our study, pregnant women with glucose threshold values measured in the first trimester, but who did not develop GDM, showed a higher percentage of neutrophils and a lower percentage of lymphocytes in the third trimester compared to the first trimester. Our study is a pilot study that included a small number of participants, in contrast to previous extensive studies that included a significantly larger number of participants. However, even with this small number of subjects, we observed a difference in neutrophils and lymphocytes in the group of women with fasting glucose disorders in the first trimester. There is no doubt that the NLR and the percentage of its components differ in healthy pregnant women and pregnant women with some pregnancy-related pathology, including GDM, considering that these are the body’s defence cells. It is important to remember that lymphocytes and neutrophils change under various inflammatory conditions and pathologies, and so they cannot be considered as good markers of GDM by themselves without other markers.

Several previous studies have highlighted the important role of T lymphocytes in the development of GDM, which is why we examined the ratios of cytotoxic and helper lymphocytes between the examined groups [34,35,36]. Our investigation revealed a statistically significant difference in the ratio of cytotoxic and helper lymphocytes in pregnant women with GDM, pregnant women with impaired fasting glucose and healthy pregnant women. A recently published study by Musumeci showed results similar to ours, demonstrating a statistically significant difference in NK cells, T lymphocytes and monocytes between pregnant women who developed GDM and healthy pregnant women. There was also a statistically significant difference in the value of IL-6 between the examined groups, in contrast to our results, in which the IL-6 values were low in all subjects and there were no statistically significant differences between them [37]. Hara et al. have claimed that the number of NK cells decreases and that the number of NKT cells increases in pregnant women with GDM, which is in accordance with our results [38].

We did not observe a difference in the percentage of B lymphocytes in either the first or third trimester in pregnant women with GDM, in the group with glucose disorder at the beginning of pregnancy or in healthy pregnant women, although a previous study reported a correlation between B lymphocytes and insulin resistance in GDM patients [39]. The previous study by Zhuang et al. included 192 subjects, of which 124 subjects had GDM. In our study, the number of subjects with GDM may have been too small to demonstrate a difference in the percentage of B lymphocytes. However, a difference in the total number of B lymphocytes does not have to exist between the examined groups, given that B lymphocytes can have both proinflammatory and anti-inflammatory effects. For this reason, in our study, the percentage of B lymphocytes that have a CD5+ cluster—which is associated with the onset of metabolic syndrome and insulin resistance—was examined. Our expectation was that the percentage of these lymphocytes would be higher in the group of pregnant women with GDM compared to the group of healthy pregnant women. While a statistically significant difference in the percentage of B lymphocytes with CD5+ cluster was not found between the groups, we observed that pregnant women with GDM had higher percentages of these lymphocytes, and we believe that a statistically significant difference would be unveiled if the study had a larger number of test subjects.

A study conducted in 969 Chinese pregnant women showed that C-peptide is a good predictive biomarker for the development of GDM [40]; however, the study in our population did not confirm this result. Similarly to insulin and insulin resistance, C-peptide values increased during the development of pregnancy in all three examined groups in our study, which is why neither C-peptide nor insulin proved to be good predictive markers of GDM. Furthermore, unlike in the previous studies—which covered the same gestational weeks of the first trimester as ours—HOMA-IR did not prove to be a good predictor of GDM in our study [41,42]. HbA1c was considered as a possible predictive biomarker for the development of GDM, and as a potential test that could replace the OGTT. However, in agreement with previous research, our research confirmed that it cannot be used, as there was no statistically significant difference in its values in the first trimester between healthy pregnant women and pregnant women who developed GDM later in pregnancy [43]. In our research, HbA1c values increased throughout pregnancy, which can be explained by the physiological changes that occur during pregnancy, i.e., increased insulin resistance and decreased haemoglobin concentration [44]. The parameter that proved to be a potential predictor of GDM was hsCRP, which had higher values in the first trimester in pregnant women who later developed GDM. Previous researchers obtained the same result; however, they also stated that additional studies are needed [45]. The values of hsCRP in the first trimester in healthy pregnant women ranged from 1.0 to 5.68 mg/L, while the values in pregnant women who later developed GDM were between 2.6 and 10.45 mg/L. Considering its non-specificity and the overlap of the intervals in which it changed in the examined groups of pregnant women, we believe that it is still not a good predictive marker of GDM. 

In our study, we did not find statistically significant differences between the examined groups in terms of proinflammatory (IL-6 and TNF-α) and anti-inflammatory cytokines (IL-10) and adiponectin. Previous studies have shown contradictory results regarding the mentioned parameters [46,47,48,49,50]. For example, a 2008 study that included 250 pregnant women showed that adiponectin values were lowered in GDM, such that it could have a predictive role, while there was no statistically significant difference in IL-6 and IL-10 between healthy pregnant women and those with GDM [48]. Reduced adiponectin in GDM, as well as increased values of TNF-α, IL-6 and anti-inflammatory IL-10 in GDM were shown in the results of Ategbo et al., who conducted their study on a group of 120 pregnant women [47]. Other studies and review articles have also pointed to different results of testing for adiponectin, IL-6, IL-10 and TNF-α, and their importance in GDM [48,49,50]. We believe that the key limitation of our research is the small number of participants, as in similar studies, due to which the data are not fully uniform and relevant.

The fatty acids are associated with the GDM pathology, given that nutritional status is an important factor before and during pregnancy. SFAs are abundant in foods and, according to several studies, they can have negative impacts on insulin sensitivity and may lead to a high risk of GDM, while PUFAs are associated with a low risk of GDM [51,52]. In our research, we confirmed that there was a difference between pregnant women with disturbed glucose metabolism and healthy pregnant women regarding PUFAs and the PUFA/SFA ratio. DHA was associated with glucose disorder but, along with AA, is essential for foetal immune system regulation and CNS development. In our study, we did not find statistically significant differences in AA and DHA between groups. Perhaps due to the small number of participants, as was the case in previous research [53], our study did not reveal MUFAs as a good GDM marker, although it is believed that supplementation with these fatty acids could contribute to glucose tolerance in pregnancy.

## 5. Conclusions

Pregnancy is a specific state of an organism in which physiological changes in the immune and metabolic profiles occur. GDM is an increasingly common pathology that occurs in pregnancy and leads to additional metabolic and immunological changes. Unfortunately, it often remains unrecognised. Screening for GDM during early pregnancy and in the second trimester of pregnancy is necessary for the timely treatment of pregnant women and the prevention of complications for both the mother and foetus. The biggest barrier in the research on potential markers for early detection of GDM and a good knowledge of immunological and metabolic changes in GDM is represented by the limitations of studies focused on pregnant women (regarding the need for a representative number of subjects and the gestational week in which the research was conducted). Future extensive research is necessary to gain better insight into the immunological and metabolic profiles of healthy pregnant women and those with GDM. In our pilot study—which included only 61 pregnant women—we observed a difference in the profiles of T lymphocytes, NK cells and PUFAs between healthy pregnant women, pregnant women with GDM and pregnant women with a fasting glucose disorder in the first trimester of pregnancy. Considering the widespread use of flow cytometry in clinical laboratories, we propose that analysing lymphocyte, NK and NKT cell profiles could be a straightforward approach for assessing the risk of GDM in pregnant women. These results can serve as a good basis for future investigations in larger numbers of subjects, which would further contribute to understanding the pathophysiology of GDM. Knowing the pathophysiology of GDM would allow for the possibility of its earlier recognition, thus promoting better treatment and reduction of complications that can cause GDM.

## Figures and Tables

**Table 1:** General characteristics and lifestyle of the participants.

	Number (%) of Participants				p *
	Control (n = 34)	GDM (n = 13)	Glucose Disturbance in First Trimester (n = 14)	Summary (n = 61)	
Age [Median (IQR)]	30 (26–33)	34 (30–36)	31 (28–36)	31 (27–34)	0.06 ^†^
Gestational week [Median (IQR)]	9 (8–11)	9 (8–11)	8 (8–10)	9 (8–11)	0.31 ^†^
BMI before pregnancy					
<19	3 (8.8)	0 (0)	2 (14.4)	5 (8.2)	0.24
19–24.9	24 (70.6)	6 (46.2)	10 (71.4)	40 (65.6)	
25–29.9	5 (14.7)	4 (30.7)	1 (7.1)	10 (16.4)	
>30	2 (5.9)	3 (23.1)	1 (7.1)	6 (9.8)	
Waist-to-hip ratio					
<0.8	15 (44.1)	6 (46.2)	7 (50)	28 (46)	0.98
0.81–0.85	9 (26.5)	3 (23.1)	4 (28.6)	16 (26)	
>0.86	10 (29.4)	4 (30.7)	3 (21.4)	17 (28)	
Physical activity					
Never	2 (6)	1 (8)	2 (14)	5 (8)	0.86
Rare	15 (44)	6 (46)	6 (43)	27 (44)	
Periodically	13 (38)	3 (23)	4 (29)	20 (33)	
Regularly	4 (12)	3 (23)	2 (14)	9 (15)	
Daily activity					
<30 min	25 (74)	8 (62)	9 (64)	42 (69)	0.66
>30 min	9 (26)	5 (38)	5 (36)	19 (31)	
Varied nutrition	31 (91)	11 (85)	14 (100)	56 (92)	0.30

IQR, interquartile range; * Fisher’s exact test; ^†^ Mann–Whitney U-test.

**Table 2:** Gynaecological and obstetric history of the participants.

	Control	GDM	Glucose Disturbance in First Trimester	Summary	p *
Age during the first menstruation [Median (IQR)]	13 (12–14) [range 11–17]	13 (12–14) [range 11–15]	13 (12–13) [range 11–14]	13 (12–14) [range 11–17]	0.29 ^†^
Menstrual cycle					0.65
Normal	7 (21)	1 (8)	3 (21)	11 (18)	
Abundant	2 (6)	0 (0)	0 (0)	2 (3)	
Scarce	4 (12)	2 (15)	0 (0)	6 (10)	
Irregular	21 (62)	10 (77)	11 (79)	42 (69)	
Spotting between cycle	1 (3)	0 (0)	1 (7)	2 (3)	0.70
PCOS	6 (18)	3 (23)	4 (29)	13 (22)	0.54
Number of births [Median (IQR)]	0 (0–1) [range 0–2]	1 (0–1) [range 0–1]	1 (0–2) [range 0–4]	1 (0–1) [range 0–4]	0.47 ^†^
Hypertension in previous pregnancy	0 (0)	1 (5)	0 (0)	1 (3)	0.23
Thyroid hormone disorders in previous pregnancy	2 (10)	3 (33)	2 (20)	7 (18)	0.35
GDM in previous pregnancy	2 (10)	3 (33)	1 (10)	6 (15)	0.32
Newborn weighs more than 4000 g in previous pregnancy	1 (5)	4 (44)	1 (10)	6 (15)	**0.03**

Bold denotes statistical significance. IQR—interquartile range; PCOS—polycystic ovary syndrome; * Fisher’s exact test; ^†^ Mann–Whitney U-test.

**Table 3:** Results of OGTT between groups.

	Median (Interquartile Range)						*p* ^†^
	Control	*p* *	GDM	*p* *	Glucose Disturbance in First Trimester	*p* *	
Fasting glucose (nmol/L)	4.45 (4.18–4.53)	**<0.001**	5.1 (4.4–6.2)	**<0.001**	4.8 (4.4–4.9)	**0.002**	**0.002**
1 h after administration of glucose (nmol/L)	6.80 (6.03–7.93)		9.0 (8.7–10.5)		7.3 (6.7–9.0)		**0.001**
2 h after administration of glucose (nmol/L)	6.05 (5.40–7.0)		8.1 (7.0–9.3)		5.8 (4.8–7.8)		**0.01**

Bold denotes statistical significance. * Friedman’s test; ^†^ Kruskal–Wallis test (post hoc Conover).

**Table 4:** Haematological, biochemical and immunological parameters in the first trimester of pregnancy.

	Median (Interquartile Range)			*p* *
	Control	GDM	Glucose Disturbance in First Trimester	
Erythrocytes (×10^12^/L)	4.28 (4.07–4.53)	4.45 (4.3–4.82)	4.46 (4.21–4.63)	0.09
Haemoglobin (g/L)	132 (123.75–138)	135 (131–142.5)	131 (115–140)	0.17
Platelets (×10^9^/L)	248.5 (204–280.25)	228 (196.5–289.5)	279 (253.25–330.5)	**0.04**
Leukocytes (×10^9^/L)	8.3 (6.68–9.5)	8 (6.95–10.45)	9.45 (7.15–9.93)	0.64
Neutrophils (%)	70.5 (66–74)	70 (65.5–76.5)	67.5 (62.75–69.5)	0.18
Lymphocytes (Ly) (%)	23 (20–28)	23 (20–26)	25 (22.5–33)	0.29
Monocytes (%)	4 (3–6)	4 (3.5–5.5)	5 (2.75–6.25)	0.83
Eosinophils (%)	1 (1–2)	2 (1–2)	2 (1–2.25)	0.78
Basophils (%)	0 (0–0.25)	0 (0–1)	0 (0–1)	0.41
Ly (%) (flow cytometry)	18 (15.75–21.25)	19 (14–22.5)	18.5 (16–21.25)	0.89
T-Ly (%) CD3+	75.55 (73.08–80.13)	75.6 (72.6–81.25)	70.3 (68.55–77.83)	0.14
T-helpers (%) CD3+CD4+	43.45 (38.93–48.43)	42.8 (39.05–51.35)	36.45 (34.6–45.5)	**0.02**
T-cytotoxic (%) CD3+CD8+	28.1 (23.13–33.03)	26.6 (22.5–31.7)	29.65 (26.4–32.13)	0.37
HELPERS/CYTOTOXIC	43.45 (38.93–48.43)	21.4 (19.53–25.68)	12.15 (11.53–15.2)	**<0.001**
NK-cells (%) CD3-CD16+56+	11.1 (7.88–14.58)	10.1 (8.65–13.75)	16.75 (13.08–21.9)	**0.03**
NKT-cells (%) CD3+CD16+56+	6.5 (4–10.28)	5 (4.15–8.5)	9.4 (4.53–10.55)	0.37
NK/NKT	1.89 (0.84–3.28)	2.26 (1.01–3.27)	2.05 (1.08–4.03)	0.96
B-Ly (%) CD19+20+	10.55 (7.78–12.2)	10.2 (7.75–13.8)	8.1 (7.35–10.48)	0.16
B-Ly subset (%) CD19+CD5+	2.35 (1.68–3.83)	2.2 (1.75–3.75)	2.05 (1.7–3.1)	0.83
Glucose (mmol/L)	4.6 (4.4–4.8)	4.8 (4.15–5.5)	5.2 (5.1–5.33)	**<0.01**
Insulin (mIU/L)	7.9 (5.05–9.63)	8.6 (6.05–10.35)	9.1 (5.45–17.28)	0.45
C-peptide (nmol/L)	0.57 (0.46–0.7)	0.63 (0.5–0.78)	0.55 (0.49–0.77)	0.61
HOMA-IR	2 (1–2)	2 (1–2.5)	2 (1–4.25)	0.33
HbA1c (mmol/mol)	33 (32–35)	35 (32–38.5)	34 (31–35.25)	0.24
CRP (mg/L)	2.75 (1.0–5.68)	4.2 (2.6–10.45)	1.45 (0.8–2.65)	**0.01**
IgG (g/L)	10.7 (9.76–12.23)	11.8 (9.46–12.9)	10.8 (9.65–12.88)	0.82
IgM (g/L)	1.09 (0.84–1.9)	1.09 (1.02–1.58)	1.46 (1.15–1.72)	0.37
IgA (g/L)	1.7 (1.34–2.28)	1.64 (1.2–2.19)	1.72 (1.42–2.2)	0.90
Iron (umol/L)	22.5 (19–27.25)	22 (17–23.5)	21.5 (16–27.25)	0.48
Ferritin (ug/L)	48 (24–70.25)	52 (43–86)	26 (19.5–44.75)	**0.02**
B12 (pmol/L)	336.5 (250.75–394.5)	352 (272.5–466)	302.5 (228.5–346.8)	0.32
Folic acid (nmol/L)	41.4 (28.45–57)	42.4 (37.85–55.3)	32.2 (29.1–57.95)	0.59
Cholesterol (mmol/L)	4.5 (3.88–4.9)	4.5 (4.2–4.9)	4.25 (3.98–4,8)	0.77
HDL Cholesterol (mmol/L)	1.8 (1.6–2.1)	1.6 (1.35–2)	1.7 (1.38–1.93)	0.12
LDL Cholesterol (mmol/L)	2.25 (1.88–2.7)	2.4 (2.25–2.8)	2.3 (2.18–2.95)	0.25
Triglycerides (mmol/L)	0.9 (0.7–1.15)	1.2 (0.95–1.5)	0.8 (0.78–1.2)	0.12
IL-6 (ng/L)	2.85 (1.5–3.58)	3.2 (1.5–5)	3.7 (2.13–4.68)	0.37
TNF-a (pg/mL)	0.4 (0.0–1.7)	0.0 (0.0–1.8)	0.0 (0.0–0.7)	0.64
IL-10 (pg/mL)	18.65 (6.07–43.15)	20.13 (0.0–53.93)	37.05 (0.00–65.29)	0.80
Adiponectin (ng/mL)	19.54 (8.6–44.6)	25.27 (12.17–37.64)	31.6 (21.3–52.6)	0.30

Bold denotes statistical significance. * Mann–Whitney U-test.

**Table 5:** Haematological, biochemical and immunological parameters in the third trimester of pregnancy.

	Median (Interquartile Range)			*p* *
	Control	GDM	Glucose Disturbance in First Trimester	
Erythrocytes (×10^12^/L)	3.89 (3.77–4.14)	4.01 (3.78–4.48)	4.04 (3.84–4.38)	0.30
Haemoglobin (g/L)	117.5 (114.75–126)	124 (118–125.5)	125 (120–129.75)	0.09
Platelets (×10^9^/L)	228.5 (200.75–267.5)	215 (184–270)	233 (192–288)	0.83
Leukocytes (×10^9^/L)	9.95 (8.43–11.25)	10.2 (7.6–12.25)	9.45 (8.48–10.4)	0.80
Neutrophils (%)	70 (66–74)	72 (68–75)	72.5 (66.75–76)	0.44
Lymphocytes (Ly) (%)	23.5 (20–27)	21 (17–25.5)	21 (17.75–25)	0.27
Monocytes (%)	5.5 (4–6)	4 (2–6)	5 (4–6.25)	0.17
Eosinophiles (%)	1 (1–2)	1 (0.5–2.5)	1 (1–2)	0.98
Basophils (%)	0 (0–1)	0 (0–0)	0 (0–1)	0.23
Ly (%) (flow cytometry)	18 (14–21)	16 (13–19)	16 (14.75–19.5)	0.42
T-Ly (%) CD3+	78.8 (75.15–83.1)	80.1 (77.7–84.1)	77.55 (71.48–81.38)	0.20
T-helpers (%) CD3+CD4+	47.35 (42.78–50.78)	46.5 (43.45–55.9)	39.65 (36.38–48.93)	0.10
T-cytotoxic (%) CD3+CD8+	29.2 (25.9–33.73)	27.8 (22.2–32.2)	30.25 (26.8–36.43)	0.50
HELPERS/CYTOTOXIC	1.69 (1.3–1.99)	1.64 (1.38–2.37)	1.28 (1.06–1.82)	0.20
NK-cells (%) CD3-CD16+56+	9.25 (7.15–12.83)	8.2 (6.55–11.35)	11.85 (10.5–19.63)	**0.03**
NKT-cells (%) CD3+CD16+56+	6.1 (4.03–8.6)	4.8 (4–9.95)	6.45 (5.18–10.95)	0.62
NK/NKT	1.42 (0.86–2.82)	1.47 (0.75–2.02)	2.07 (0.61–3.35)	0.71
B-Ly (%) CD19+20+	8.8 (6.68–12)	9.2 (6.05–12.85)	7.95 (6.28–11.63)	0.76
B-Ly subset (%) CD19+CD5+	1.65 (0.98–2.48)	1.4 (0.95–2.5)	1.8 (0.9–2.2)	0.97
Glucose (mmol/L)	4.45 (4.3–4.8)	4.8 (4.4–5.3)	4.7 (4.45–4.93)	0.06
Insulin (mIU/L)	11.15 (8.1–15.13)	12.7 (9.1–19.3)	12.85 (9.1–16.85)	0.48
C-peptide (nmol/L)	0.81 (0.7–1.04)	1.02 (0.71–1.5)	0.78 (0.67–1.06)	0.30
HOMA-IR	2 (2–3)	3 (2–4)	3 (2–3.25)	0.39
HbA1c (mmol/mol)	35 (32.75–37)	37 (33–40.5)	34.5 (30–37.25)	0.24
CRP (mg/L)	3.6 (2.1–5.83)	6.4 (2.45–9.15)	2.7 (1.28–4.03)	**0.04**
IgG (g/L)	8.11 (7.26–9.75)	9.5 (7.76–10.95)	8.78 (7.16–9.95)	0.46
IgM (g/L)	1.01 (0.82–1.96)	1.15 (0.94–1.52)	1.17 (0.96–1.47)	0.96
IgA (g/L)	1.73 (1.14–2.03)	1.5 (1.16–1.93)	1.73 (1.37–2.04)	0.82
Iron (umol/L)	13 (8.75–16)	15 (11–21)	20 (12.25–23.75)	**0.03**
Ferritin (ug/L)	14 (11–21.5)	16 (9.5–23)	23.5 (12.75–30.25)	0.14
B12 (pmol/L)	213.5 (173–279)	223 (209.5–311)	217 (144.5–260.5)	0.58
Folic acid (nmol/L)	29.4 (18.83–42.68)	33.6 (14.9–58.5)	41.7 (32.43–60.55)	0.19
Cholesterol (mmol/L)	6.8 (6.18–7.93)	6.4 (5.95–7.45)	7.55 (6.53–8.43)	0.26
HDL cholesterol (mmol/L)	2 (1.7–2.33)	1.9 (1.55–2.25)	1.85 (1.58–2.33)	0.70
LDL cholesterol (mmol/L)	4.1 (3.58–5.05)	3.7 (3.05–4.5)	4.65 (4.05–5.55)	0.07
Triglycerides (mmol/L)	2.4 (2.08–3)	3 (2.5–3.45)	2.5 (2.25–3.23)	0.11
IL-6 (ng/L)	2.7 (1.88–4.4)	2.65 (1.98–3.45)	2.9 (1.65–3.7)	0.75
TNF-a (pg/mL)	0.4 (0.0–1.7)	0.2 (0.0–1.6)	0.0 (0.0–0.8)	0.20
IL-10 (pg/mL)	12.21 (0.40–20.71)	14.7 (0.0–105.4)	33.21 (4.33–484.28)	0.40
Adiponectin (ng/mL)	16.5 (4.56–37.4)	7.77(3.06–101.4)	16.91 (10.26–43.13)	0.60

Bold denotes statistical significance. * Mann–Whitney U-test.

**Table 6:** Fatty acid results in first trimester of pregnancy.

	Median (Interquartile Range)			*p* *
	Control	GDM	Glucose Disturbance in First Trimester	
SFAs (%)	46.7 (41.67–53.00)	46.49 (42.3–53.18)	44.92 (40.94–49.57)	0.06
MUFAs (%)	22.68 (17.37–29.80)	24.1 (21.41–26.51)	23.49 (19.17–26.62)	0.64
PUFAs (%)	**30.23 (25.51–35.34)**	29.41 (22.72–35.75)	**31.93 (26.70–35.26)**	**0.04**
UFAs (%)	53.30 (47.00–58.30)	53.51 (46.82–57.70)	55.08 (50.43–59.51)	0.06
UFAs/SFAs (%)	1.14 (0.89–1.40)	1.15 (0.88–1.36)	1.23 (1.02–1.47)	0.06
PUFAs/SFAs (%)	**0.64 (0.53–0.81)**	0.62 (0.43–0.85)	**0.71 (0.57–0.87)**	**0.03**
AA/DHA (%)	0.21 (0.09–0.71)	0.23 (0.12–0.54)	0.19 (0.06–0.58)	0.39
AA/EPA (%)	0.19 (0.09–0.93)	0.21 (0.07–0.45)	0.16 (0.06–0.60)	0.56
EPA/DHA (%)	1.02 (0.59–2.72)	1.27 (0.44–2.62)	1.08 (0.65–1.78)	0.76

Bold denotes statistical significance. SFAs—saturated fatty acids; MUFAs—monounsaturated fatty acids; PUFAs—polyunsaturated fatty acids; UFAs—unsaturated fatty acids; AA—arachidonic acid; DHA—docosahexaenoic acid; EPA—eicosapentaenoic acid. * Mann–Whitney U-test.

## Data Availability

The data that support finding of this study are available from the corresponding author upon reasonable request.

## References

[1] Omazić J., Viljetić B., Ivić V., Kadivnik M., Zibar L., Müller A., Wagner J. (2021). Early markers of gestational diabetes mellitus: What we know and which way forward?. Biochem. Med..

[2] Wang H., Li N., Chivese T., Werfalli M., Sun H., Yuen L., Hoegfeldt C.A., Powe C.E., Immanuel J., Karuranga S. (2022). IDF Diabetes Atlas: Estimation of Global and Regional Gestational Diabetes Mellitus Prevalence for 2021 by International Association of Diabetes in Pregnancy Study Group’s Criteria. Diabetes Res. Clin. Pract..

[3] Semnani-Azad Z., Gaillard R., Hughes A.E., Boyle K.E., Tobias D.K., Perng W. (2024). Precision stratification of prognostic risk factors associated with outcomes in gestational diabetes mellitus: A systematic review. Commun. Med..

[4] Mestman J.H. (2002). Historical Notes on Diabetes and Pregnancy. Endocrinologist.

[5] Pons R.S., Rockett F.C., De Almeida Rubin B., Oppermann M.L.R., Bosa V.L. (2015). Risk factors for gestational diabetes mellitus in a sample of pregnant women diagnosed with the disease. Diabetol. Metab. Syndr..

[6] Mao L., Gao B., Chang H., Shen H. (2024). Interaction and Metabolic Pathways: Elucidating the Role of Gut Microbiota in Gestational Diabetes Mellitus Pathogenesis. Metabolites.

[7] Catalano P.M., Huston L., Amini S.B., Kalhan S.C. (1999). Longitudinal changes in glucose metabolism during pregnancy in obese women with normal glucose tolerance and gestational diabetes mellitus. Am. J. Obstet. Gynecol..

[8] Weir G.C., Laybutt D.R., Kaneto H., Bonner-Weir S., Sharma A. (2001). Beta-cell adaptation and decompensation during the progression of diabetes. Diabetes.

[9] Andersson-Hall U., Carlsson N.G., Sandberg A.S., Holmäng A. (2018). Circulating Linoleic Acid is Associated with Improved Glucose Tolerance in Women after Gestational Diabetes. Nutrients.

[10] Pinto J., Almeida L.M., Martins A.S., Duarte D., Barros A.S., Galhano E., Pita C., Almeida M.D.C., Carreira I.M., Gil A.M. (2015). Prediction of Gestational Diabetes through NMR Metabolomics of Maternal Blood. J. Proteome Res..

[11] Boyson J.E., Aktan I., Barkhuff D.A., Chant A. (2008). NKT cells at the maternal-fetal interface. Immunol. Investig..

[12] Lv X., Gao Y., Dong T., Yang L. (2018). Role of Natural Killer T (NKT) Cells in Type II Diabetes-Induced Vascular Injuries. Med. Sci. Monit..

[13] McElwain C.J., McCarthy F.P., McCarthy C.M. (2021). Gestational Diabetes Mellitus and Maternal Immune Dysregulation: What We Know So Far. Int. J. Mol. Sci..

[14] Miko E., Barakonyi A., Meggyes M., Szereday L. (2021). The Role of Type I and Type II NKT Cells in Materno-Fetal Immunity. Biomedicines.

[15] Metzger B.E., Gabbe S.G., Persson B., Buchanan T.A., Catalano P.A., Damm P., Dyer A.R., de Leiva A., Hod M., International Association of Diabetes and Pregnancy Study Groups Consensus Panel (2010). International association of diabetes and pregnancy study groups recommendations on the diagnosis and classification of hyperglycemia in pregnancy. Diabetes Care.

[16] Carlson R.V., Boyd K.M., Webb D.J. (2004). The revision of the Declaration of Helsinki: Past, present and future. Br. J. Clin. Pharmacol..

[17] Folch J., Lees M., Sloane Stanley G.H. (1957). A simple method for the isolation and purification of total lipides from animal tissues. J. Biol. Chem..

[18] Wang Y., Wu P., Huang Y., Yang X., Sun F., Ye Y., Lai Y., Ouyang J., Wu L., Li Y. (2022). BMI and lipidomic biomarkers with risk of gestational diabetes in pregnant women. Obesity.

[19] Miao M., Dai M., Zhang Y., Sun F., Guo X., Sun G. (2017). Influence of maternal overweight, obesity and gestational weight gain on the perinatal outcomes in women with gestational diabetes mellitus. Sci. Rep..

[20] Rahnemaei F.A., Abdi F., Kazemian E., Shaterian N., Shaterian N., Behesht Aeen F. (2022). Association between body mass index in the first half of pregnancy and gestational diabetes: A systematic review. SAGE Open Med..

[21] Mirabelli M., Tocci V., Donnici A., Giuliano S., Sarnelli P., Salatino A., Greco M., Puccio L., Chiefari E., Foti D.P. (2023). Maternal Preconception Body Mass Index Overtakes Age as a Risk Factor for Gestational Diabetes Mellitus. J. Clin. Med..

[22] Levent Cirakli Z., Gulec N. (2022). Evaluation of White Blood-cell-based Inflammatory Markers in Gestational Diabetes Mellitus. Bakirkoy Tip Derg..

[23] Zhang Y., Zhang Y., Zhao L., Shang Y., He D., Chen J. (2021). Distribution of complete blood count constituents in gestational diabetes mellitus. Medicine.

[24] Fashami M.A., Hajian S., Afrakhteh M., Khoob M.K. (2020). Is there an association between platelet and blood inflammatory indices and the risk of gestational diabetes mellitus?. Obstet. Gynecol. Sci..

[25] Yang H., Zhu C., Ma Q., Long Y., Cheng Z. (2015). Variations of blood cells in prediction of gestational diabetes mellitus. J. Perinat. Med..

[26] Sissala N., Mustaniemi S., Kajantie E., Vääräsmäki M., Koivunen P. (2022). Higher hemoglobin levels are an independent risk factor for gestational diabetes. Sci. Rep..

[27] Mehrabian F., Hosseini S.M. (2013). Comparison of gestational diabetes mellitus and pre-eclampsia in women with high hemoglobin in the first trimester of pregnancy: A longitudinal study. Pak. J. Med. Sci..

[28] Ye Y.-X., Wu P., Yang X., Wu L., Lai Y., Ouyang J., Li Y., Li P., Hu Y., Wang Y.-X. (2023). Blood Cell Parameters from Early to Middle Pregnancy and Risk of Gestational Diabetes Mellitus. J. Clin. Endocrinol. Metab..

[29] Sahin M., Oguz A., Tüzün D., Işiktaş O., Işiktaş S., Ülgen C., Şahin H., Gul K. (2022). A new marker predicting gestational diabetes mellitus: First trimester neutrophil/lymphocyte ratio. Medicine.

[30] Sargin M.A., Yassa M., Taymur B.D., Celik A., Ergun E., Tug N. (2016). Neutrophil-to-lymphocyte and platelet-to-lymphocyte ratios: Are they useful for predicting gestational diabetes mellitus during pregnancy?. Ther. Clin. Risk Manag..

[31] Pace N.P., Vassallo J. (2021). Association Between Neutrophil-Lymphocyte Ratio and Gestational Diabetes—A Systematic Review and Meta-Analysis. J. Endocr. Soc..

[32] Sun X., Sun H., Li P. (2021). Association of circulating inflammatory cells and platelets with gestational diabetes and pregnancy outcomes. Clin. Chim. Acta.

[33] Sun T., Meng F., Zhao H., Yang M., Zhang R., Yu Z., Huang X., Ding H., Liu J., Zang S. (2020). Elevated First-Trimester Neutrophil Count Is Closely Associated with the Development of Maternal Gestational Diabetes Mellitus and Adverse Pregnancy Outcomes. Diabetes.

[34] Arain H., Patel T., Mureanu N., Efthymiou A., Lombardi G., Tree T., Nicolaides K.H., Shangaris P. (2023). Regulatory T cells in the peripheral blood of women with gestational diabetes: A systematic review and meta-analysis. Front. Immunol..

[35] Sheu A., Chan Y., Ferguson A., Bakhtyari M.B., Hawke W., White C., Chan Y.F., Bertolino P.J., Woon H.G., Palendira U. (2018). A proinflammatory CD4+ T cell phenotype in gestational diabetes mellitus. Diabetologia.

[36] Sifnaios E., Mastorakos G., Psarra K., Panagopoulos N.-D., Panoulis K., Vitoratos N., Rizos D., Creatsas G. (2019). Gestational Diabetes and T-cell (Th1/Th2/Th17/Treg) Immune Profile. In Vivo.

[37] Musumeci A., McElwain C.J., Manna S., McCarthy F., McCarthy C. (2024). Exposure to Gestational Diabetes Mellitus increases subclinical inflammation mediated in part by obesity. Clin. Exp. Immunol..

[38] Hara C.d.C.P., França E.L., Fagundes D.L.G., de Queiroz A.A., Rudge M.V.C., Honorio-França A.C., Calderon I.d.M.P. (2016). Characterization of Natural Killer Cells and Cytokines in Maternal Placenta and Fetus of Diabetic Mothers. J. Immunol. Res..

[39] Zhuang Y., Zhang J., Li Y., Gu H., Zhao J., Sun Y., Wang R., Zhang C., Chen W., Weng J. (2019). B Lymphocytes Are Predictors of Insulin Resistance in Women with Gestational Diabetes Mellitus. Endocr. Metab. Immune Disord.-Drug Targets.

[40] Yang X., Ye Y., Wu P., Lu Q., Liu Y., Yuan J., Song X., Yan S., Qi X., Wang Y.-X. (2022). Association between early-pregnancy serum C-peptide and risk of gestational diabetes mellitus: A nested case–control study among Chinese women. Nutr. Metab..

[41] Song S., Zhang Y., Qiao X., Duo Y., Xu J., Peng Z., Zhang J., Chen Y., Nie X., Sun Q. (2022). HOMA-IR as a risk factor of gestational diabetes mellitus and a novel simple surrogate index in early pregnancy. Int. J. Gynaecol. Obstet..

[42] Duo Y., Song S., Zhang Y., Qiao X., Xu J., Zhang J., Peng Z., Chen Y., Nie X., Sun Q. (2022). Predictability of HOMA-IR for Gestational Diabetes Mellitus in Early Pregnancy Based on Different First Trimester BMI Values. J. Pers. Med..

[43] Valadan M., Bahramnezhad Z., Golshahi F., Feizabad E. (2022). The role of first-trimester HbA1c in the early detection of gestational diabetes. BMC Pregnancy Childbirth.

[44] Edelson P.K., E James K., Leong A., Arenas J., Cayford M., Callahan M.J., Bernstein S.N., Tangren J.S., Hivert M.-F., Higgins J.M. (2020). Longitudinal Changes in the Relationship between Hemoglobin A1c and Glucose Tolerance across Pregnancy and Postpartum. J. Clin. Endocrinol. Metab..

[45] Amirian A., Rahnemaei F.A., Abdi F. (2020). Role of C-reactive Protein(CRP) or high-sensitivity CRP in predicting gestational diabetes Mellitus:Systematic review. Diabetes Metab. Syndr..

[46] Georgiou H.M., Lappas M., Georgiou G.M., Marita A., Bryant V.J., Hiscock R., Permezel M., Khalil Z., Rice G.E. (2008). Screening for biomarkers predictive of gestational diabetes mellitus. Acta Diabetol..

[47] Atègbo J.-M., Grissa O., Yessoufou A., Hichami A., Dramane K.L., Moutairou K., Miled A., Grissa A., Jerbi M., Tabka Z. (2006). Modulation of Adipokines and Cytokines in Gestational Diabetes and Macrosomia. J. Clin. Endocrinol. Metab..

[48] Brink H.S., Van Der Lely A.J., Van Der Linden J. (2016). The potential role of biomarkers in predicting gestational diabetes. Endocr. Connect..

[49] Li S., Yang H. (2019). Relationship between advanced glycation end products and gestational diabetes mellitus. J. Matern.-Fetal Neonatal Med..

[50] Ni J., Wang Y., Zhu J., Zheng T., Yu J., Xiong Y. (2022). Interleukin-10 levels and risk of gestational diabetes mellitus: A systematic review and meta-analysis. J. Matern. Fetal. Neonatal. Med..

[51] Sun Z., Deng Z., Wei X., Wang N., Yang J., Li W., Wu M., Liu Y., He G. (2022). Circulating saturated fatty acids and risk of gestational diabetes mellitus: A cross-sectional study and meta-analysis. Front. Nutr..

[52] Du H., Li D., Molive L.M., Wu N. (2024). Advances in free fatty acid profiles in gestational diabetes mellitus. J. Transl. Med..

[53] Tsoi K.Y., Zhu Y., Wu J., Sun Q., Hinkle S.N., Li L.-J., Chen Z., Weir N.L., Tsai M.Y., Ma R.C. (2022). Plasma Phospholipid Monounsaturated Fatty Acids and Gestational Diabetes Mellitus: A Longitudinal Study in the NICHD Fetal Growth Studies–Singletons Cohort. Diabetes.

